# Limited 12-hour pharmacokinetic assessment of CBD and CBDA isolates compared to their full-spectrum extracts in healthy adult beagles

**DOI:** 10.3389/fvets.2025.1639846

**Published:** 2025-08-12

**Authors:** Tongxin Charlotte Wang, Joseph J. Wakshlag, Alex Lyubimov, Alexander Zakharov, Beatriz Gomez, Wayne Schwark, Nathalie L. Trottier

**Affiliations:** ^1^Department of Animal Science, Cornell University, Ithaca, NY, United States; ^2^Department of Clinical Sciences, College of Veterinary Medicine, Cornell University, Ithaca, NY, United States; ^3^Toxicology Research Laboratory, Department of Pharmacology, University of Illinois at Chicago, Chicago, IL, United States; ^4^Department of Molecular Medicine, Cornell University, Ithaca, NY, United States

**Keywords:** pharmacokinetic, CBD, CBDA, cannabinoids, canine, safety

## Abstract

**Introduction:**

The rapidly expanding market for therapeutic cannabinoid products has intensified research on their safety and efficacy in pets. Full-spectrum hemp extracts contain compounds such as terpenoids and flavonoids that may act synergistically via the “entourage effect,” yet their application in companion animals remains underexplored. This study assessed the pharmacokinetics and safety of isolated and full-spectrum cannabinoids in dogs.

**Methods:**

Eight healthy adult Beagle dogs (four males and four females) were randomly assigned to a 4 × 4 Latin square design (two dogs per kennel, same sex), consisting of four experimental periods and four treatments: CBD isolate (1 mg/kg), CBDA isolate (1 mg/kg), CBDA full spectrum (FS) (1 mg/kg), and a combined CBD/CBDA FS (1 mg/kg). Treatments were administered twice daily (every 12 h). In the morning, dogs received their assigned treatment following their daily ration of dry kibble and were immediately offered 122 grams of wet food. Each experimental period lasted 1 week and was followed by a three-week washout period.

**Results:**

No adverse events were associated with any treatment. CBDA showed higher Cmax and AUC than CBD in both isolate and FS forms (*p* < 0.001). CBDA in CBD/CBDA FS had a shorter Tmax compared to CBD (*p* = 0.019). Mean residence time and elimination half-life did not differ among treatments.

**Conclusion:**

CBDA demonstrated superior absorption compared to CBD. No evidence supported enhanced absorption from full-spectrum products, suggesting the “entourage effect” may involve receptor-level interactions rather than absorption. All treatments were well tolerated, with normal CBC and chemistry results, indicating that administering CBD or CBDA, either as isolates or in full-spectrum extracts, at 1 mg/kg every 12 h for 1 week is safe in healthy adult dogs. This is the first comprehensive comparison of full-spectrum, isolate, and acidic cannabinoid forms in dogs.

## 1 Introduction

In companion animal medicine, cannabinoids have gained significant interest over the past decade. In 2018, Epidiolex, a hemp-based CBD isolate drug for epilepsy in young children, received FDA approval and was successfully launched on the market, demonstrating safety for long-term use (1, 2). Both cannabinoid isolates and full-spectrum CBD-rich products have also been evaluated in animals for safety and pharmaceutical applications (3, 4). In dogs diagnosed with idiopathic epilepsy, CBD- and CBDA-rich hemp products have been associated with reduced seizure frequency (5). According to a recent published review article (3), five human studies have shown that CBD can alleviate pain, thereby improving quality of life and allowing for reduced dosage of other medications as part of a multimodal pain management approach.

Additionally, seven other studies have reported beneficial effects of CBD/CBDA-based nutraceuticals in treating canine osteoarthritis (3). However, data on the absorption, retention, and clinical efficacy of various cannabinoid form in dogs remain limited. To date, only three studies (6–8) have investigated the safety and tolerability of acidic cannabinoids such as CBDA in dogs. While some publications have addressed the efficacy of full hemp extract, comprehensive data on full-spectrum cannabinoids are still lacking. Preclinical studies in rodents and humans suggest that differences may exist in the absorption kinetics of CBD and CBDA when delivered as isolates versus full-spectrum hemp products containing other minor cannabinoids (9, 10). A thorough understanding of the pharmacokinetics and safety profiles of CBD and CBDA in dogs is essential before developing evidence-based guidelines for their use in veterinary medicine.

The objectives of this research were twofold: first, to determine the pharmacokinetics of full-spectrum cannabinoid products versus isolates (CBD and CBDA) and limited metabolite production in adult beagle dogs; and second, to assess the safety and tolerability of both cannabinoid forms, the isolates and the full-spectrum formulations (CBD isolate oil, CBDA isolate oil, full-spectrum CBDA oil, and a full-spectrum CBD/CBDA mixture) in healthy adult beagles. We hypothesized that, compared to CBD, acidic cannabinoids (CBDA) and full-spectrum (FS) cannabinoids would exhibit greater absorption in adult dogs.

## 2 Materials and methods

### 2.1 Animals and study design

All experimental procedures were approved by the Cornell University Animal Care and Use Committee (protocol #2022–0211).

Eight healthy adult Beagles (four males and four females), all 2 years old and weighing between 7.5 and 11.3 kg, were used in this study. The dogs were owned by the Cornell University College of Veterinary Medicine teaching dog colony. Prior to the study, same-sex dogs were housed in groups of four in separate temperature-controlled rooms maintained at 26°C. For the study, dogs were re-paired and housed in pairs within 2 × 5 m kennels, with two kennels per room. Dogs were acclimated to the new housing arrangement for 2 days before the experimental procedures began.

Kennels were randomly assigned to a 4 × 4 Latin square design with four experimental periods and four treatments: CBD isolate (1 mg/kg), CBDA isolate (1 mg/kg), CBDA FS (1 mg/kg), and CBD/CBDA FS (1 mg/kg). Treatments were administered twice daily (every 12 hours). In the morning, dogs received their assigned treatment following their daily ration of dry kibble and were immediately offered 122 grams of wet food. Each experimental period lasted 1 week and was followed by a three-week washout period. At the beginning of each experimental period, dogs were weighed to calculate the appropriate dosage of cannabinoids per treatment. The initial body weight (mean kg ± standard deviation) across treatment groups were 9.27 ± 1.33 (CBD isolate), 9.26 ± 1.35 (CBDA isolate), 9.40 ± 1.48 (CBDA FS) and 9.44 ± 1.44 (CBD/CBDA FS).

All dogs were fed dry food (LabDiet 5 L18, PMI Nutrition International, Brentwood, MO, USA) once a day in the morning (06.00). During each experimental period, treatments were administered directly into the dogs’ mouth using a 1-mL Luer-slip type syringe. Following dosing, each dog received an additional portion of their daily dietary allocation consisting of 122 grams of wet food (Purina Pro Plan Savory Chicken and Rice Formula, Nestle Purina, St. Louis, MO) in the morning (07.00) and the evening (19.00), immediately after treatments throughout the study.

### 2.2 Cannabinoid products

Isolates of CBD and CBDA (Open Book Extracts, Roxboro, NC) were each emulsified in sesame oil to produce 30 mg/mL suspensions. A full hemp extract (CBDA FS) obtained from Cultivate Biologics (Louisville, CO) was emulsified in sesame seed oil to yield a suspension containing 25 mg/mL of CBDA, 1.1 mg/mL THCA, 1.2 mg/mL CBCA, 0.5 mg/mL CBGA, 0.1 mg/mL CBDVA, and 1.1 mg/mL CBD. Concentrations of other cannabinoids were below the limits of detection. A full-spectrum hemp extract (CBD/CBDA FS) from Cultivate Biologics (Louisville, CO) was emulsified in sesame oil to yield a suspension of 28.2 mg/mL CBD, 27 mg/mL CBDA, 0.5 mg/mL CBG, 0.4 mg/mL CBGA, 1.9 mg/mL THC, 0.6 mg/mL THCA, 1.4 mg/mL CBC, and 1.7 mg/mL CBCA, with other cannabinoids being below the limits of detection. All extracts were tested every 2 weeks and showed less than 5% variation from the original screening, indicating stability.

### 2.3 Data collection

**Figure 1:**
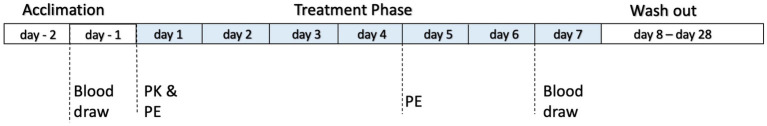
Timeline of each study period. PK, pharmacokinetics; PE, physical examination.

On day 1, pharmacokinetic (PK) sampling was conducted over a 12-h period, with blood samples (2 mL) collected at 0.5, 1, 2, 4, 8, and 12 h post oral dosing. From days 2 to 7, dogs received twice-daily dosing at 12-h intervals. Blood samples (6 mL) were collected 1 day prior to the start of the study and 6 h after the morning dosing on day 7 to assess hepatic chemistry and serum cannabinoid profiles. A timeline of this study is presented in Figure 1. The PK sampling was conducted over a limited 12-h time window, as the highest observed concentration (Cmax), the time point to reach maximum concentration (Tmax), and the area under the concentration–time curve (AUC), representing total systemic exposure, were the primary parameters used to assess absorption rate and extent. This design was informed by prior literature indicating that absorption occurs within 2 h in many studies involving dogs. An abbreviated PK protocol was therefore implemented, with careful consideration given to dog welfare. Physical examinations were performed on days 1 and 5 at 1 and 4 h post dosing, with a primary focus on identifying any neurological adverse events.

For blood sample collection, dogs were manually restrained. Either cephalic or jugular vein was used, and blood was transferred into serum tubes for the analysis of cannabinoids and hepatic serum chemistry. Samples were allowed to coagulate for 30–60 min, at room temperature, followed by centrifugation at 1,700 × *g* for 6 min. Serum was then aliquoted: one 2-mL aliquot was submitted for clinical biochemistry, and two 0.5-mL aliquots were immediately frozen at −80°C for subsequent cannabinoid profile analysis.

### 2.4 Physical exam

Prior to enrollment in the study (day 0), physical examinations were conducted to evaluate the dogs’ health. On days 1 and 5, dogs were examined at 1 and 4 h post dosing. Heart rate was measured and neurologic functions, including hopping proprioception response, protrusion of the nictitating membrane, and palpebral reflex, were assessed as potential adverse events in dogs (11, 12). Signs of lethargy, vomiting, and diarrhea were also closely monitored.

### 2.5 Sample analyses

#### 2.5.1 Complete blood count and serum chemistry

Blood samples for complete blood counts and serum biochemistry profile were submitted to Cornell University Diagnostic Laboratory Clinical Pathology service for analyses.

#### 2.5.2 Serum cannabinoids

Analysis of cannabinoids in dog serum was performed at the Toxicology Research Laboratory, University of Illinois at Chicago, as described previously for various species (6, 13–16), with the modification that 6-OH-CBD was added with a reference analyte, as shown in Supplementary Table 1.

#### 2.5.3 Pharmacokinetics

The non-compartmental 12-h pharmacokinetic analysis for each hemp-derived cannabinoid (CBD, CBDA, CBG, CBGA, Δ9-THC, THCA) was performed utilizing a commercial software system (PK solutions 2.0, Summit PK, Montrose, CO). Semi-log plots were utilized to determine linearity of the elimination profiles. The results generated were time to maximal concentrations (Tmax), maximum serum concentration (Cmax), elimination half-life (T 12), area under the curve to the last time-point (AUC_0–12_), and mean residence time (MRT). The program predicts steady state average serum concentrations (Css Ave) based on the assumption that steady state levels are achieved after 5 half-lives with selected frequencies of administration.

### 2.6 Statistical analysis

Statistical analysis of PK parameters was performed using R version 4.3.2 (RStudio, PBC, Boston, MA, USA). Pharmacokinetic parameters, including Cmax, Tmax, T½ elimination, AUC and MRT, were assessed for normality using the Shapiro–Wilk test. As the data were not normally distributed (*p* < 0.05), all PK parameters were log-transformed prior to statistical analysis. Treatment effects were evaluated using a linear mixed-effects model, with period and treatment as fixed effects and dog and kennel as random effects to account for individual and housing variability.

Maximum serum concentration, Tmax, and AUC were calculated using data from all eight dogs. However, in several cases, CBD or CBDA blood concentrations were insufficient to estimate T1/2 el, MRT, and Css. Values for those parameters were not available for one dog in the CBD isolate treatment, three dogs in the CBDA isolate treatment, three dogs in the CBDA FS treatment, three dogs in the CBD component of the CBD/CBDA FS treatment, and four dogs in the CBDA component of the CBD/CBDA FS treatment. To address our specific hypotheses, treatment comparisons were pre-planned prior to data collection and analysis, as follows: CBD isolate vs. CBD in CBD/CBDA FS, CBDA isolate vs. CBDA in CBD/CBDA FS, CBD isolate versus CBDA isolate, and CBDA isolate versus CBDA FS. Statistical significance was set at *p* < 0.05. Differences in CBD and CBDA serum concentrations on day 7 in the respective primary treatment groups were evaluated. Paired *T*-test test was used to determine the difference for CBD between CBD isolate and CBD/CBDA FS, and Friedman test was conducted to determine the difference in CBDA between CBDA isolate, CBDA FS and CBDA/CBD FS.

Differences in 12-h serum cannabinoid metabolite concentrations between CBD isolate and CBDA/CBD FS groups were evaluated. Following confirmation of normal data distribution with Shapiro–Wilk test, Welch’s *t*-tests were used to compare concentrations between CBD isolate and CBDA/CBD FS groups for each metabolite.

## 3 Results

### 3.1 Twelve-hour pharmacokinetics

Pharmacokinetic data and *p*-values are presented in Tables 1, 2. For Cmax, CBDA isolate had a higher peak serum concentration compared to CBD isolate (235.51 ± 65.59 vs. 69.80 ± 35.44 ng/mL, *p* < 0.001). CBDA in CBD/CBDA FS had a higher Cmax than CBD in the same formulation (229.30 ± 145.51 vs. 64.66 ± 37.05 ng/mL, *p* < 0.001). No other treatment comparisons differed for Cmax.

**Table 1:** Pharmacokinetics of CBD and CBDA in dogs administrated with CBD isolate, CBDA isolate, CBDA FS, CBD/CBDA FS oil^1^.

Treatment						Treatment effect *p*-value^4^
Item	CBD isolate	CBDA isolate	CBDA FS	CBD^2^	CBDA^3^	
				CBD/CBDA FS		
*n*	8	8	8	8	8	
Cmax^5^ (ng/mL)	69.80 ± 35.44	235.51 ± 65.59	208.28 ± 66.05	64.66 ± 37.05	229.30 ± 145.51	*
Tmax^6^ (h)	2.75 ± 2.31	1.75 ± 2.58	0.94 ± 0.68	3.75 ± 2.71	1.81 ± 2.55	*
T1/2 el^7^^,^^11^ (h)	3.76 ± 1.22	6.19 ± 3.06	6.63 ± 2.69	4.5 ± 3.46	4.68 ± 1.97	ns
AUC^8^ (ng-h/mL)	420.61 ± 45.52	1255.70 ± 463.52	997.59 ± 366.4	354.45 ± 124.65	1240.97 ± 450.55	*
MRT^9^^,^^11^ (h)	5.73 ± 1.88	NA^12^	NA^13^	7.04 ± 4.77	7.35 ± 3.06	ns
Css^10^^,^^11^ (ng/ml)	42.83 ± 4.62	NA^12^	NA^13^	51.10 ± 14.92	108.90 ± 32.48	*

1 Values are means ± standard deviation.

2 Pharmacokinetics of CBD in the CBD/CBDA full spectrum treatment.

3 Pharmacokinetics of CBDA in the CBD/CBDA full spectrum treatment.

4 *p*-values for specific treatment comparisons are presented in Table 2.

5 Maximum serum concentration.

6 Time of maximum concentration.

7 Half-life of elimination.

8 Area under the serum concentration curve (0 to 12 hours).

9 Mean residence time.

10 Steady state serum concentration.

11 Three dogs are missing due to low concentrations.

12 For CBDA isolate treatment, values for MRT and Css were not available due to low concentrations.

13 For CBDA full spectrum treatment, values for MRT and Css were not available due to low concentrations.

**Table 2:** *p*-value of specific treatment comparisons of the pharmacokinetics for dogs administrated with CBD isolate, CBDA isolate, CBDA FS, CBD/CBDA FS oil^1^.

Item	CBD in CBD/CBDA FS^1^ vs CBD isolate	CBDA in CBD/CBDA FS vs CBDA isolate	CBDA FS vs CBDA isolate	CBDA isolate vs CBD isolate	CBDA in CBD/CBDA FS vs CBD in CBD/CBDA FS
*n*	8	8	8	8	8
Cmax^2^ (ng/mL)	*p* = 0.979	*p* = 0.949	*p* = 0.945	*p* < 0.001	*p* < 0.001
Tmax^3^ (h)	*p* = 0.799	*p* = 0.999	*p* = 0.918	*p* = 0.120	*p* = 0.019
T1/2 el^4^ (h)	*p* = 0.999	*p* = 0.944	*p* = 0.997	*p* = 0.402	*p* = 0.962
AUC^5^ (ng-h/mL)	*p* = 0.627	*p* = 1.000	*p* = 0.635	*p* < 0.001	*p* < 0.001
MRT^6^ (h)	*p* = 0.989	NA^8^	NA^8^	NA^8^	*p* = 0.940
Css^7^ (ng/ml)	*p* = 0.972	NA^8^	NA^8^	NA^8^	*p* = 0.012

1 Values are calculated based on log transformed data.

2 Maximum concentration.

3 Time of maximum concentration.

4 Half-life of elimination. Three dogs were removed from the model due to insufficient concentration.

5 Area under the serum concentration curve (0 to 12 hours).

6 Mean residence time.

7 Steady state serum concentration.

8 Data not available.

For Tmax, CBDA in CBD/CBDA FS reached peak concentration earlier than CBD in the same formulation (1.81 ± 2.55 vs. 3.75 ± 2.71 h, *p* = 0.019). All other Tmax comparisons showed no differences.

The elimination half-life did not differ among any treatments. For AUC, CBDA isolate had a higher AUC than CBD isolate (1255.70 ± 463.52 vs. 420.61 ± 45.52 ng·h/mL, *p* < 0.001). CBDA in CBD/CBDA FS had a higher AUC than CBD in the same formulation (1240.97 ± 450.55 vs. 354.45 ± 124.65 ng·h/mL, *p* < 0.001). No other AUC comparisons differed.

Mean residence time and steady-state serum concentrations of CBD did not differ between CBD/CBDA FS and CBD isolate groups. Steady-state serum concentration of CBDA was higher than CBD in the CBD/CBDA FS group (108.9 ± 32.48 vs. 51.1 ± 14.92 ng/mL, *p* = 0.012).

On day 7, serum concentrations of CBD, CBDA, THC, and THCA were measured (Supplementary Figure 1). CBD and CBDA concentrations did not differ between treatments (*p* = 0.392). Trace amounts of THC and THCA were detected, primarily in the CBD/CBDA FS and CBDA FS groups.

Serum concentrations of the metabolites 6-OH-CBD and 7-COOH-CBD are presented in Supplementary Figures 2–4. No differences were observed between treatments for 6-OH-CBD or 7-COOH-CBD (*p* = 0.578 and *p* = 0.788, respectively). In the CBD/CBDA FS group, 7-COOH-CBD serum concentrations peaked at approximately 4 h post-dose. In the CBD isolate group, peak concentrations occurred between 4 and 8 h. Serum 6-OH-CBD concentrations in the CBD isolate group peaked at approximately 2 h and declined gradually. In the CBD/CBDA FS group, peak 6-OH-CBD concentrations were reached around 2 h, followed by a steady decline over 12 h.

### 3.2 Complete blood counts and serum chemistry

Mean and standard deviations of CBC values on days 1 and 7 are presented in Supplementary Tables 2, 3. All values were within reference ranges. Blood chemistry profiles are summarized in Supplementary Tables 4, 5. Urea nitrogen and phosphate levels were above reference ranges in all treatments. For the blood chemistry profile on days 1 and 7 (Supplementary Tables 4, 5), across all groups, urea nitrogen and phosphate levels were closer to the higher end of the reference range, while total protein and globulin were closer to the lower end. These variations were likely related to food intake timing and hydration status, and are considered clinically irrelevant, as multiple other studies have demonstrated the safety of long-term exposure. Total protein and globulin levels were below reference ranges. All other parameters of electrolyte balance, renal function, liver function, muscle function, and mineral homeostasis were within reference ranges.

### 3.3 Physical examination and health data

Physical examination data, including heart rate and body weight, are presented in Supplementary Table 6. Throughout the duration of the study, no signs of neurological changes such as pupil dilation, protrusion of the nictitating membrane, or proprioceptive delays were observed. Vomiting was noted in two groups of dogs (CBDA in FS, CBD/CBDA in FS) on day two of the fourth period following the evening dose. Vomiting observed in these two groups may have been due to cumulative gastrointestinal sensitivity or mild irritation associated with repeated dosing over multiple periods. The sesame seed oil formulation could also have contributed to gastric upset, especially when administered in the evening, when gastric emptying is slower. Notably, the absence of other changes in appetite or increased incidence of diarrhea suggests that this effect was transient and formulation-related rather than indicative of systemic intolerance or broader gastrointestinal dysfunction.

## 4 Discussion

The cannabis plant contains more than 400 compounds, including over 120 phytocannabinoids, more than 140 terpenoids, as well as various flavonoids and alkaloids. Cannabigerolic acid (CBGA) is the precursor to other major cannabinoids including Δ9-tetrahydrocannabinol (THC), cannabidiol (CBD), and cannabichromene (17). While many of these compounds have yet to be fully characterized, isolated forms such as THC and CBD have been well-studied in medicinal research. Although several studies have investigated the safety and tolerability of the decarboxylated forms of cannabinoids, particularly CBD in dogs, data on the safety and tolerability of full-spectrum and acidic forms remain limited (5, 6, 13, 17, 18).

In the cannabis plant, cannabinoids and other compounds function synergistically as secondary metabolites serving as antioxidants and contributing to the plant’s defense against predators (18). Following purification, cannabinoids can be present in various formats, including as isolates of single cannabinoids either in their decarboxylated or acidic forms, as full spectrum extracts preserving the plant’s native cannabinoid and terpene profile, or as mixtures containing two or more cannabinoids. The term “entourage effect” refers to the enhance overall impact when cannabinoids are used in combination with other minor compounds, e.g., terpenoids and flavonoids (9).

For the acidic form, pharmacokinetic studies have demonstrated its safety when administered to rabbits, horses, cats, dogs, humans and parrots (13–15, 19–21), with all of those studies suggesting superior absorption of CBDA (6, 13, 14, 17–19). Prior data from human pharmacokinetics have shown that CBD absorption appears to be enhanced when a portion of CBDA is delivered together (10). Further examination of rodents (mice) has suggested the CBDA absorption is relatively poor as an isolate, and the absorption was nearly 14 fold better while delivering in a full spectrum mixture. It was proposed that the other minor cannabinoids allowed for inhibition of enterocyte efflux enzymes allowing for better absorption as the primary mechanism (9). Therefore it is expected that there may be differing effects of full spectrum products compared to isolates which has been reported in the human literature. Full-spectrum cannabinoids are suggested to enhance symptom relief when treating mood or anxiety disorders, as well as seizures (22). Although we found little evidence to support that the major cannabinoids CBD and CBDA were enhanced by administrated as full spectrum products, there appears to be a numerical trend over 7 days on full spectrum CBDA absorption, therefore the “entourage effect” is not likely to be related to the major cannabinoid absorption, but rather to the minor components found in full spectrum products. However, our data is limited, and additional studies are needed to determine these concentrations at steady state.

Pharmacokinetic data that compares full spectrum cannabinoids to isolate cannabinoid forms was unknown in dogs prior to this study. The objectives of this study were to (1) determine the pharmacokinetics of full-spectrum hemp extract and cannabinoid isolates including quantification of CBD metabolites (6-OH-CBD, 7-COOH-CBD) in healthy adult dogs, and to (2) assess the tolerability and safety of orally dosed cannabinoid isolates (CBD, CBDA) and a hemp extract in adult beagle dogs. It was hypothesized that, compared to the isolate cannabinoid (CBD), acidic cannabinoids (CBDA) and full-spectrum cannabinoids would exhibit greater absorption in adult dogs. No significant changes in complete blood count (CBC) and serum chemistry profiles were anticipated during the one-week period of cannabinoid administration.

Earlier studies have reported that the oral bioavailability of cannabinoids in dogs is low due to extensive first-pass metabolism (23, 24). However, transdermal administration of cannabinoids has not resulted in serum concentrations comparable to those achieved with oral dosing (25). Due to the lipophilic nature of cannabinoids, and the stratum corneum, their absorption through the skin epithelium is limited (16).

In our study, the absorption of orally dosed CBD isolate at 1 mg/kg BW was low (69.8 ± 35.44 ng/mL) and comparable to that of other studies. Gamble et al. reported Cmax value of 102 ng/mL for CBD dosed at 1 mg/kg BW in a long chain triglyceride oil vehicle format to fasted dogs. Wakshlag et al. reported Cmax values of 145 ± 69 and 124 ± 62 ng/mL, respectively, for CBD administered at the same dose (1 mg/kg BW) formulated with either a mixture of medium chain triglycerides (MCT) and a sesame oil or a mixture of sunflower lecithin and sesame oil. Della Rocca et al. reported a Cmax of 206.77 ± 167.07 ng/mL for CBD administered at 1 mg/kg BW in MCT oil. In contrast, Vaughn et al. reported a significantly lower Cmax 30 ± 7 ng/mL for the same CBD dose and carrier oil. In contrast, in the studies by Deabold et al. and Wakshlag et al., when CBD was dosed at 1 mg/kg using an enriched soft chew, Cmax was greater, with values of 301 ± 63 ng/mL and 226 ± 89 ng/mL, respectively. Therefore, the type of carrier vehicle to dose CBD appears to be a driving factor in CBD absorption. In addition to carrier types, various absorption of cannabinoids were observed in other species with fasted animals or fed before dosing (13, 20). In our study, dogs were fed dry kibble before dosing, and wet food immediately after. At the same dosage (1 mg/kg), the CBD AUC observed in this study (420.61 ± 45.52 ng-h/mL) was comparable to that reported by Della Roca et al. (536.05 ng-h/mL) using MCT oil as carrier vehicle. In Doran et al., feeding dogs before dosing showed a twofold increase in AUC at 5, 10, 20 mg/kg dosage. Although additional feeding did not lead to a higher AUC in our study, feeding state and encapsulation materials used could be the other driving factors when considering cannabinoids absorption (3, 26).

In our study, Cmax, AUC, and Css for CBDA isolates were greater than those observed for the CBD isolate. These findings support previous research in dogs, cats, horses and steers, suggesting that the acidic form of cannabinoids exhibit superior absorption compared to their decarboxylated counterparts in mammalian species (7, 14, 15, 19). Similar to results reported by Gamble et al., co-administration of CBDA and CBD (each at 1 mg/kg) did not enhance the Cmax of CBD (99 ± 29.13 ng/mL). Likewise, in our study, the presence of CBDA in the CBD/CBDA FS mixture did not influence the Cmax of CBD when compared to the CBD isolate (64.66 ± 37.05 ng/mL vs. 69.8 ± 35.44 ng/mL, respectively). In contrast, Tittle et al. reported higher Cmax values for CBD in dogs dosed with a CBD-rich hemp extract formulated as either a soft gel (267.6 ± 98.9 ng/mL) or in sesame oil (184.5 ± 55.8 ng/mL), with both formulations containing CBD and CBDA at 1 mg/kg. This discrepancy may indicate that the absorption of acidic cannabinoids is less affected by feeding status compared to their decarboxylated counterparts.

The Cmax and AUC values for CBDA in CBD/CBDA FS were greater compared to those for CBD in CBD/CBDA FS. This is the first study that compares CBD and CBDA pharmacokinetics in a full spectrum mixture. This finding aligns closely with the CBDA isolate treatment, which had greater absorption compared to CBD isolate treatment. CBDA has a low affinity for both CB1 or CB2, is one of the minor cannabinoids that functions as allosteric regulators for 5-HT1α receptors, and reported to be 1,000 time more potent than CBD in binding to these receptor sites (27). CBDA also showed better anti-hyperalgesia, anti-nausea and anti-seizure effects in rodent models (18). Those findings in the rodent studies align with the previous studies in dogs, cats and horses (7, 15, 21, 28), suggesting a better potency in the cannabinoid acidic form.

The Cmax for CBDA in the CBD/CBDA FS mixture remained unaffected with the addition of CBD, other minor cannabinoids, terpenes and flavonoids in the mixture, compared to the CBDA isolate (229.30 ± 145.51 vs. 235.51 ± 65.59) and similarly for Tmax, AUC and Css. When comparing the CBDA FS to CBDA isolate, no differences between these treatments were observed, similar to CBD results, suggesting that FS formulations play little role in the absorption and or/retention of CBD or CBDA.

The time-course serum concentrations of two primary CBD metabolites 6-OH-CBD and 7-COOH-CBD (Supplementary Figures 2–4) indicate potential treatment-dependent differences in metabolite levels. Interpretation of these data should be made with caution, as several time points were below the detection limit, and proper statistical analyses could not be conducted. In Supplementary Figure 2, dogs receiving the CBD isolate for 1 week appeared to show slightly higher 6-OH-CBD levels than those receiving the CBD/CBDA FS treatment, with peak concentration of 6-OH-CBD for both groups occurring between 2 and 4 h. Furthermore, 6-OH-CBD appeared higher than the traditional metabolite 7-COOH-CBD, as supported by previous studies in dogs (3), suggesting that dogs undergo differential metabolism of CBD when compared to humans and horses. These results are consistent with the known first-pass hepatic metabolism, where 6-OH-CBD serves as a primary oxidative metabolite that rapidly forms and either clears through renal excretion or undergoes further unidentified metabolism for biliary excretion (29). Moreover, prior pharmacokinetic studies has shown that dogs exhibit only 1–2% of the 7-COOH-CBD levels observed in humans receiving similar dosages (3, 7).

Further research is warranted to evaluate the biological activity and potency of 6-OH-CBD as a minor metabolite in dogs. In addition, CBDA metabolism has not been thoroughly investigated with only one study in extrahepatic microsomes suggesting that there is direct glucuronidation of CBDA at the acidic carboxylic acid (30). These findings reinforce that cannabinoid metabolism is sensitive to formulation, feeding status, and co-administered compounds, and that species-specific differences in CBD metabolism are likely substantial.

While metabolic differences were observed between treatments, it is important to also consider how the presence of additional cannabinoids and terpenes may influence the pharmacological effects of CBD through the proposed entourage effect. The entourage effect was first suggested in a preclinical study in 1998 when describing metabolites interactions (31). This term was later widely used in the field of medicinal cannabinoids, as the example of polypharmacy (use of multiple drugs to treat a single condition), e.g., full spectrum cannabinoids. The potential entourage effect can be variable (32). One of the common misleading concepts is that the entourage effects are often described as beneficial synergistic effects, while adverse effects, either caused by antagonism or additive effects are often ignored, leading to a public awareness of the beneficial sides only of the entourage effect (33). While many studies have emphasized the entourage effect in both humans and animals, limited research has been done to provide robust evidence. The pharmacokinetic results of this study do not support a better absorption of either CBD or CBDA when provided in a full spectrum. This may be due to the industrial purification methods, or that the specific cannabis chemovars used in this study did not contain the effective terpenoids or flavonoids or proportions of minor cannabinoids to promote enhanced absorption.

In our study, no adverse events were associated with a 7-day administration of CBD or CBDA isolates, or of these cannabinoids in various combinations and full spectrum. There was no elevated serum ALT, which has been observed in previous studies in cats and dogs (5, 25) nor the more innocuous ALP enzyme in this study. However, to thoroughly understand the mechanisms and hepatic safety behind the entourage effect, additional research is needed.

The results of this study are the first of a comprehensive comparison between full-spectrum, isolate, and acidic forms of cannabinoids. The study further supports that CBDA, the acidic form of CBD, is absorbed better in dogs. Previous research in mouse suggests that CBDA interacts with transporter breast cancer resistance protein (BCRP), and that CBG and THC inhibit BCRP-mediated transport of CBDA, thus leading to an elevated plasma concentration for CBDA (9). In our study, we found no evidence that full-spectrum cannabinoids have enhanced absorption in dogs. Therefore, there is a need for further validation with isolate CBDA and addition of CBG to test out the entourage effect in the canine model, as it relates to receptor biology rather than cannabinoids promoting synergistic absorption. Based on the physical assessment, the lack of adverse events, and CBC and chemical profiles falling within reference ranges, CBD, CBDA, and these isolates combined or presented in full spectrum are safe for dogs when provided at 1 mg/kg every 12 h during a one-week treatment protocol.

Due to the complex variations in cannabis chemovars, the findings are only applicable to the specific formulations tested in this study. Additionally, due to the limitations of the small sample size and the short duration of the trial, the generalizability remains limited. Future studies with larger sample sizes, different ages and breeds, and longer trial durations are necessary before providing comprehensive guidelines for cannabinoid usage in veterinary medicine.

## Data Availability

The raw data supporting the conclusions of this article will be made available by the authors, without undue reservation.

## Supplementary material

SUPPLEMENTARY FIGURE 1 Average serum concentrations of major cannabinoids on day 7 by treatment group. Values represent means ± standard error of the mean. One dog was excluded from the CBD isolate group due to unsuccessful dosing.

SUPPLEMENTARY FIGURE 2 Serum 6-OH-CBD and 7-COOH-CBD metabolite concentrations on day 7 in dogs receiving CBD isolate and CBD/CBDA FS. One dog was excluded from the CBD isolate group due to unsuccessful dosing.

SUPPLEMENTARY FIGURE 3 Serum concentration of 7-COOH-CBD over 12 hours. Values represent means ± standard error of the mean.

SUPPLEMENTARY FIGURE 4 Serum concentration of 6-OH-CBD over 12 hours. Values represent means ± standard error of the mean.
