# Supplementary figures and images for: Limited 12-hour pharmacokinetic assessment of CBD and CBDA isolates compared to their full-spectrum extracts in healthy adult beagles
Source: Front Vet Sci. 2025 Aug 12;12:1639846. doi: 10.3389/fvets.2025.1639846 (PMC12379728; doi:10.3389/fvets.2025.1639846)

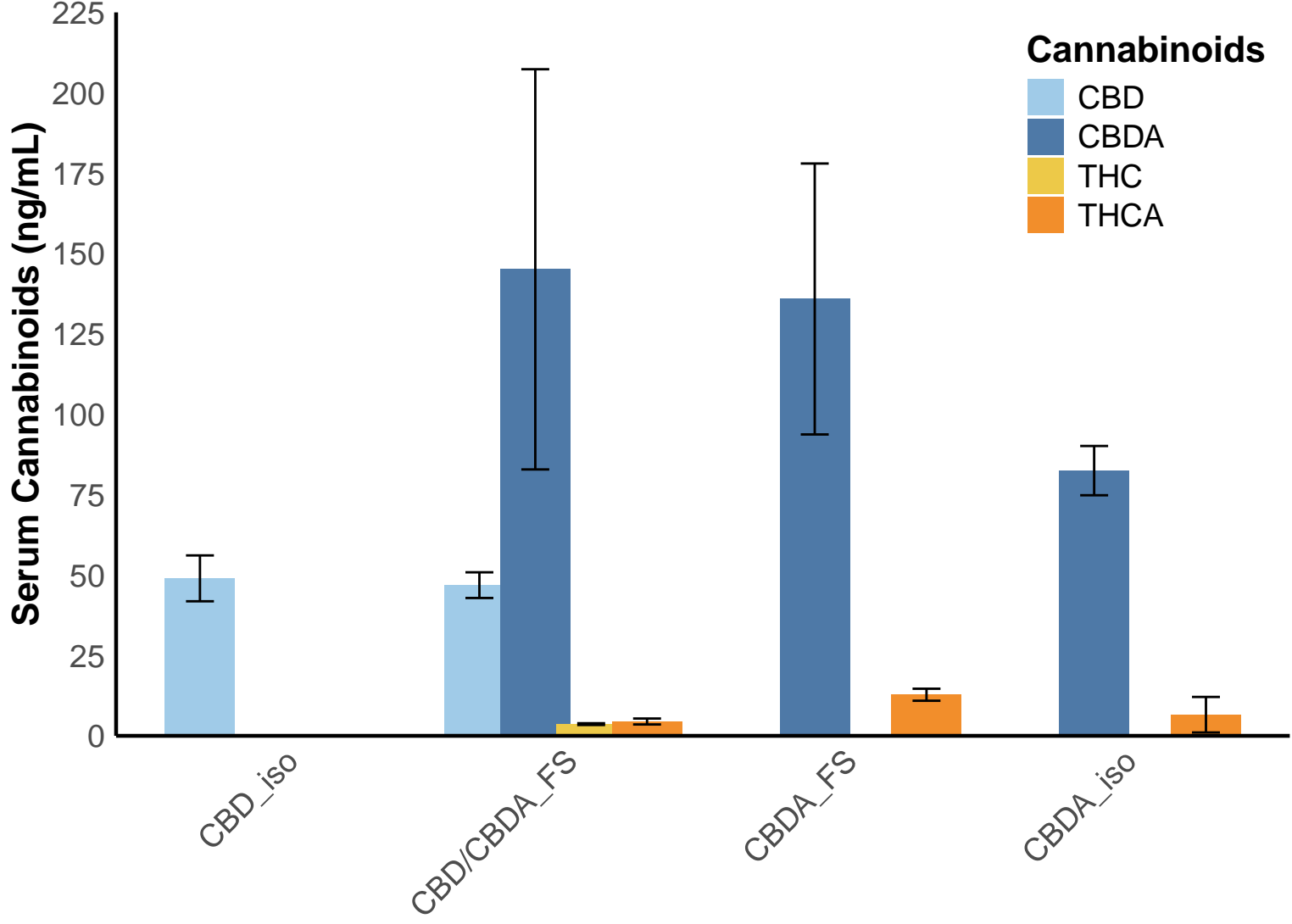

Supplement: SUPPLEMENTARY FIGURE 1 — Average serum concentrations of major cannabinoids on day 7 by treatment group. Values represent means ± standard error of the mean. One dog was excluded from the CBD isolate group due to unsuccessful dosing. [file Image_1.pdf]

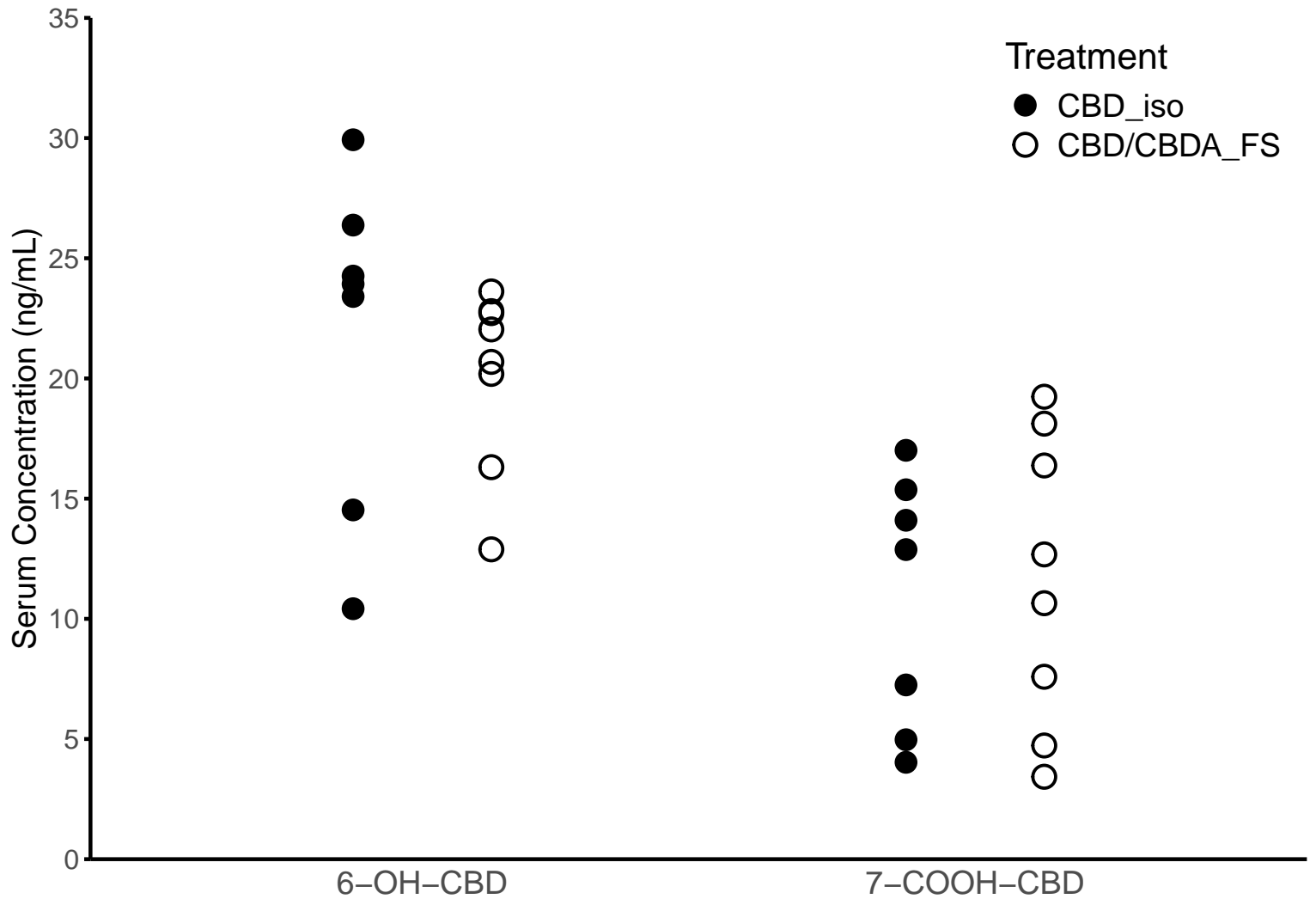

Supplement: SUPPLEMENTARY FIGURE 2 — Serum 6-OH-CBD and 7-COOH-CBD metabolite concentrations on day 7 in dogs receiving CBD isolate and CBD/CBDA FS. One dog was excluded from the CBD isolate group due to unsuccessful dosing. [file Image_2.pdf]

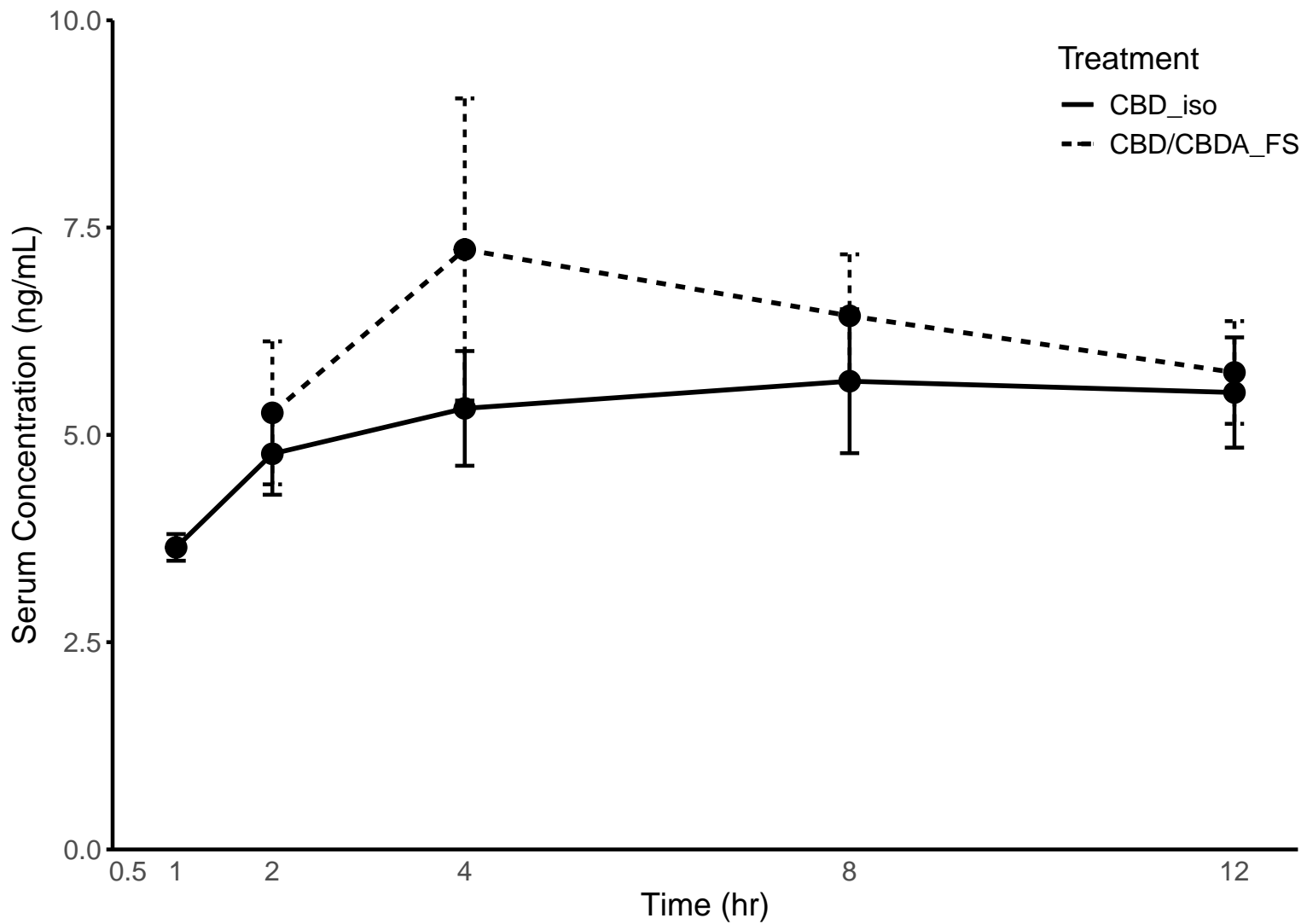

Supplement: SUPPLEMENTARY FIGURE 3 — Serum concentration of 7-COOH-CBD over 12 hours. Values represent means ± standard error of the mean. [file Image_3.pdf]

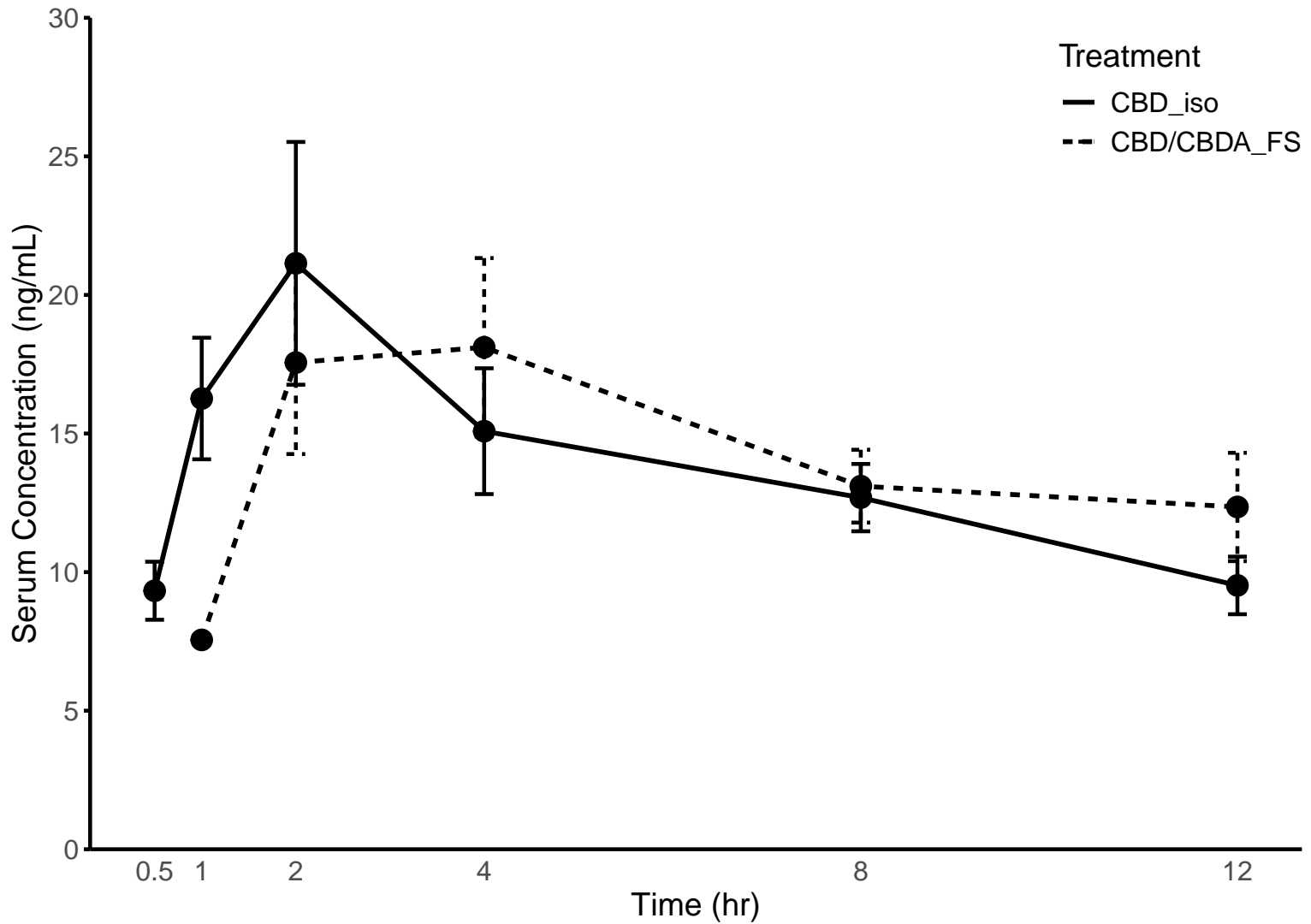

Supplement: SUPPLEMENTARY FIGURE 4 — Serum concentration of 6-OH-CBD over 12 hours. Values represent means ± standard error of the mean. [file Image_4.pdf]
