# Supplementary material for: Limited 12-hour pharmacokinetic assessment of CBD and CBDA isolates compared to their full-spectrum extracts in healthy adult beagles
Source: Front Vet Sci. 2025 Aug 12;12:1639846. doi: 10.3389/fvets.2025.1639846 (PMC12379728; doi:10.3389/fvets.2025.1639846)
Supplement: Supplementary file 5 [file Table_1.docx]

Supplemental Table 1. LC-MS/MS parameters and calibration curve range in dog serum.

| Reference Standard | | | Internal Standard | | Calibration Curve Range (ng⋅mL^-1^) |
| --- | --- | --- | --- | --- | --- |
| Name | Retention Time (min) | MRM (Polarity) | Name | MRM (Polarity) |  |
| CBD | 4.7 | 315>193 (+) | CBD-d3 | 318>196 (+) | 2.5 – 2,500 |
| CBDA | 4.4 | 357>245 (-) | CBDA-d3 | 360>248 (-) | 2.5 – 5,000 |
| THC | 5.8 | 315>193 (+) | THC-d3 | 318>196 (+) | 2.5 – 1,000 |
| THCA | 6.3 | 357>313 (-) | THCA-d3 | 360>316 (-) | 0.5 – 1,000 |
| CBG | 4.6 | 317>193 (+) | CBG-d3 | 320>196 (+) | 1 – 1,000 |
| CBGA | 4.5 | 361>219 (+) | CBGA-d3 | 364>222 (+) | 1 – 1,000 |
| CBC | 6.1 | 315>193 (+) | THC-d3 | 318>196 (+) | 5 – 1,000 |
| CBN | 5.4 | 311>223 (+) | CBD-d3 | 318>196 (+) | 2.5 – 1,000 |
| 7-COOH-CBD | 2.4 | 345>299 (+) | 7-COOH-CBD-d3 | 348>302 (+) | 2.5 – 1,000 |
| 7-OH-CBD | 2.5 | 313>201 (+) | 7-OH-CBD-d5 | 318>201 (+) | 5 – 1,000 |
| 6-OH-CBD | 2.3 | 313>201 (+) | 7-OH-CBD-d5 | 318>201 (+) | 5 – 1,000 |
| COOH-THC | 3.7 | 345>299 (+) | COOH-THC-d9 | 354>308 (+) | 2.5 – 500 |
| COOH-THC-Glu | 2.2 | 521>345 (+) | COOH-THC-d9 | 354>308 (+) | 2.5 – 500 |
| 11-OH-THC | 3.6 | 331>201 (+) | 11-OH-THC-d3 | 334>201 (+) | 5 – 1000 |

| Supplemental Table 2. Complete blood count profile on day 1 after oral administration of CBD isolate, CBDA isolate, CBDA FS and CBD/CBDA FS^1^. | | | | | | |
| --- | --- | --- | --- | --- | --- | --- |
| Treatment |  | CBD isolate | CBDA isolate | CBDA FS | CBD/CBDA FS |  |
| Items | Reference range | Day 1 | Day 1 | Day 1 | Day 1 |  |
| Hematocrit | 41 - 58  % | 53.12 ± 2.59 (49.00 - 56.00) | 53.62 ± 3.16  (48.00 - 59.00) | 53.00 ± 4.81  (46.00 - 60.00) | 53.25 ± 3.85  (47.00 - 57.00) |  |
| Hemoglobin | 14.1 - 20.1  g/dL | 17.64 ± 0.91  (16.20 - 18.80) | 17.77 ± 0.97  (16.10 - 19.20) | 17.54 ± 1.45  (15.20 - 19.40) | 17.54 ± 1.09  (15.70 - 18.80) |  |
| RBC | 5.7 - 8.5  mill/uL | 7.40 ± 0.40 (6.70 - 8.00) | 7.45 ± 0.33  (7.20 - 8.00) | 7.35 ± 0.48  (6.50 - 8.00) | 7.34 ± 0.34  (6.80 - 7.90) |  |
| WBC | 5.7 - 14.2  thou/uL | 7.20 ± 1.20  (5.70 - 9.20) | 7.22 ± 1.08  (6.10 - 9.30) | 7.41 ± 1.38  (6.00 - 9.60) | 7.55 ± 1.44  (5.70 - 9.20) |  |
| Segmented Neutrophils | 3.0 - 9.6  thou/uL | 4.38 ± 0.78  (3.60 - 5.70) | 4.28 ± 1.00 (2.80 - 6.20) | 4.22 ± 0.88  (3.10 - 5.50) | 4.45 ± 1.00 (3.40 - 5.80) |  |
| Lymphocytes | 1.1 - 4.5  thou/uL | 2.39 ± 0.49  (1.80 - 2.90) | 2.45 ± 0.42  (1.70 - 3.00) | 2.67 ± 0.49  (1.90 - 3.40) | 2.54 ± 0.52  (1.80 - 3.20) |  |
| Monocytes | 0.1 - 1.0  thou/uL | 0.28 ± 0.09  (0.20 - 0.40) | 0.30 ± 0.16  (0.20 - 0.60) | 0.25 ± 0.09  (0.10 - 0.40) | 0.32 ± 0.21  (0.20 - 0.80) |  |
| Eosinophils | 0.1 - 2.1  thou/uL | 0.15 ± 0.09  (0.00 - 0.30) | 0.16 ± 0.05 (0.10 - 0.20) | 0.22 ± 0.10 (0.10 - 0.40) | 0.19 ± 0.11  (0.10 - 0.40) |  |
| Basophils | 0.0 - 0.1  thou/uL | 0.00 ± 0.00 (0.00 - 0.00) | 0.01 ± 0.04  (0.00 - 0.10) | 0.03 ± 0.05  (0.00 - 0.10) | 0.01 ± 0.04  (0.00 - 0.10) |  |
| Platelet Count | 186 - 545  thou/uL | 224.88 ± 46.47  (158.00 - 297.00) | 233.50 ± 54.80  (140.00 - 305.00) | 238.00 ± 52.84  (159.00 - 309.00) | 245.00 ± 47.67  (160.00 - 310.00) |  |
| ^1^Values are means ± standard deviation of the mean. Values in parentheses represent the range across all dogs (i.e., the minimum to the maximum value). | | | | | | |

| Supplemental Table 3. Complete blood count profile on day 7 after oral administration of CBD isolate, CBDA isolate, CBDA full spectrum and CBD/CBDA full spectrum^1^. | | | | | | | |
| --- | --- | --- | --- | --- | --- | --- | --- |
| Treatment |  | CBD isolate | CBDA isolate | CBDA FS | | CBD/CBDA FS | |
| Items | Reference range | Day 7 | Day 7 | Day 7 | | Day 7 | |
| Hematocrit | 41 - 58  % | 52.25 ± 2.82  (49.00 - 57.00) | 51.75 ± 4.37 (45.00 - 58.00) | | 51.50 ± 4.07  (47.00 - 58.00) | | 53.25 ± 2.12  (50.00 - 56.00) |
| Hemoglobin | 14.1 - 20.1  g/dL | 17.51 ± 0.67  (16.60 - 18.40) | 17.32 ± 1.41  (15.30 - 19.20) | | 17.32 ± 1.22  (16.00 - 19.00) | | 17.81 ± 0.70 (16.60 - 18.60) |
| RBC | 5.7 - 8.5  mill/uL | 7.38 ± 0.22  (7.10 - 7.60) | 7.28 ± 0.43  (6.80 - 8.00) | | 7.26 ± 0.46  (6.60 - 8.00) | | 7.49 ± 0.23  (7.00 - 7.70) |
| WBC | 5.7 - 14.2  thou/uL | 8.81 ± 2.43  (6.60 - 14.20) | 7.88 ± 1.33  (6.10 - 10.00) | | 7.95 ± 1.55  (6.30 - 10.20) | | 8.16 ± 1.22  (6.20 - 10.10) |
| Segmented Neutrophils | 3.0 - 9.6  thou/uL | 5.42 ± 1.79  (3.90 - 9.40) | 4.70 ± 0.74  (3.90 - 5.90) | | 4.79 ± 0.98  (3.50 - 6.20) | | 4.76 ± 0.74  (3.50 - 5.80) |
| Lymphocytes | 1.1 - 4.5  thou/uL | 2.75 ± 0.48  (2.10 - 3.60) | 2.62 ± 0.55  (1.70 - 3.30) | | 2.54 ± 0.89  (0.60 - 3.40) | | 2.89 ± 0.63  (2.20 - 4.10) |
| Monocytes | 0.1 - 1.0  thou/uL | 0.31 ± 0.22  (0.10 - 0.70) | 0.29 ± 0.08  (0.20 - 0.40) | | 0.36 ± 0.16  (0.20 - 0.70) | | 0.28 ± 0.07  (0.20-0.40) |
| Eosinophils | 0.1 - 2.1  thou/uL | 0.26 ± 0.13  (0.10 - 0.50) | 0.20 ± 0.11  (0.00 - 0.30) | | 0.25 ± 0.12  (0.00 - 0.40) | | 0.24 ± 0.12  (0.00 - 0.40) |
| Basophils | 0.0 - 0.1  thou/uL | 0.01 ± 0.04  (0.00 - 0.10) | 0.01 ± 0.04  (0.00 - 0.10) | | 0.01 ± 0.04  (0.00 - 0.10) | | 0.00 ± 0.00 (0.00 - 0.00) |
| Platelet Count | 186 - 545  thou/uL | 236.25 ± 44.81  (148.00 - 299.00) | 246.50 ± 61.84  (140.00 - 325.00) | | 237.25 ± 55.71  (144.00 - 306.00) | | 240.25 ± 39.47  (188.00 - 297.00) |
| ^1^Values are means ± standard deviation of the mean. Values in parentheses represent the range across all dogs (i.e., the minimum to the maximum value). | | | | | | | |

| Supplemental Table 4. Chemistry profile on day 1 after oral administration of CBD isolate, CBDA isolate, CBDA FS and CBD/CBDA FS^1^. | | | | | | |
| --- | --- | --- | --- | --- | --- | --- |
| Treatment |  | CBD isolate | CBDA isolate | CBDA FS | CBD/CBDA FS |  |
| Items | Reference range | Day 1 | Day 1 | Day 1 | Day 1 |  |
| Sodium | 143.00 - 150.00  mEq/L | 146.50 ± 1.31  (144.00 - 148.00) | 146.50 ± 1.41  (145.00 - 149.00) | 146.75 ± 0.71  (146.00 - 148.00) | 146.86 ± 0.69  (146.00 - 148.00) |  |
| Potassium | 4.10 - 5.40  mEq/L | 4.69 ± 0.16  (4.40 - 4.90) | 4.73 ± 0.18  (4.40 - 4.90) | 4.95 ± 0.32  (4.60 - 5.50) | 4.86 ± 0.27  (4.40 - 5.20) |  |
| Chloride | 106.00 - 114.00  mEq/L | 112.13 ± 2.30  (109.00 - 116.00) | 112.13 ± 2.03  (110.00 - 115.00) | 113.00 ± 1.31  (110.00 - 114.00) | 113.29 ± 1.50  (111.00 - 115.00) |  |
| Bicarbonate | 14.00 - 24.00  mEq/L | 20.25 ± 2.25  (17.00 - 24.00) | 19.63 ± 1.51  (17.00 - 22.00) | 20.25 ± 2.19  (18.00 - 24.00) | 19.71 ± 1.60  (17.00 - 22.00) |  |
| Urea Nitrogen | 9.00 - 26.00  mg/dL | 26.50 ± 2.62  (23.00 - 30.00) | 27.25 ± 2.19  (24.00 - 30.00) | 27.25 ± 2.76  (22.00 - 31.00) | 27.57 ± 3.36  (23.00 - 33.00) |  |
| Creatinine | 0.60 - 1.40  mg/dL | 0.80 ± 0.09  (0.60 - 0.90) | 0.84 ± 0.13  (0.60 - 1.00) | 0.84 ± 0.09  (0.70 - 0.90) | 0.84 ± 0.11  (0.70 - 1.00) |  |
| Calcium | 9.40 - 11.10  mg/dL | 10.36 ± 0.26  (10.00 - 10.90) | 10.33 ± 0.27  (10.00 - 10.70) | 10.29 ± 0.20  (9.90 - 10.60) | 10.29 ± 0.17  (10.10 - 10.60) |  |
| Phosphate | 2.70 - 5.40  mg/dL | 4.84 ± 0.76  (3.70 - 5.90) | 5.14 ± 0.54  (4.10 - 5.80) | 4.81 ± 0.42  (4.10 - 5.30) | 4.81 ± 0.62  (4.20 - 5.80) |  |
| Magnesium | 1.50 - 2.10  mEq/L | 1.60 ± 0.15  (1.40 - 1.90) | 1.63 ± 0.09  (1.50 - 1.80) | 1.59 ± 0.06  (1.50 - 1.70) | 1.60 ± 0.10  (1.50 - 1.80) |  |
| Total Protein | 5.50 - 7.20  g/dL | 5.56 ± 0.24  (5.20 - 5.90) | 5.60 ± 0.16  (5.40 - 5.90) | 5.48 ± 0.19  (5.10 - 5.70) | 5.43 ± 0.19  (5.10 - 5.70) |  |
| Albumin | 3.20 - 4.10  g/dL | 3.64 ± 0.18  (3.50 - 40) | 3.61 ± 0.15  (3.50 - 3.90) | 3.53 ± 0.07  (3.40 - 3.60) | 3.60 ± 0.13  (3.50 - 3.80) |  |
| Globulin | 1.90 - 3.70  g/dL | 1.93 ± 0.22  (1.70 - 2.40) | 1.99 ± 0.22  (1.70 - 2.30) | 1.95 ± 0.21  (1.60 - 2.30) | 1.83 ± 0.15  (1.60 - 2.00) |  |
| Glucose | 68.00 - 104.00  mg/dL | 90.63 ± 8.77  (76.00 - 107.00) | 94.00 ± 17.86  (64.00 - 115.00) | 86.88 ± 10.84  (73.00 - 105.00) | 94.29 ± 5.91  (88.00 - 105.00) |  |
| ALT | 17.00 - 95.00  U/L | 28.38 ± 7.19  (21.00 - 45.00) | 29.63 ± 8.05  (20.00 - 46.00) | 27.13 ± 6.73  (22.00 - 43.00) | 27.14 ± 7.45  (20.00 - 42.00) |  |
| AST | 18.00 - 56.00  U/L | 26.50 ± 4.72  (21.00 - 35.00) | 26.75 ± 3.99  (23.00 - 36.00) | 25.13 ± 4.22  (19.00 - 31.00) | 26.00 ± 3.21  (22.00 - 32.00) |  |
| Alkaline Phosphatase | 7.00 - 115.00  U/L | 45.63 ± 12.69  (26.00 - 65.00) | 46.50 ± 13.92  (29.00 - 76.00) | 46.88 ± 14.14  (29.00 - 68.00) | 49.71 ± 18.87  (29.00 - 89.00) |  |
| GGT | 0.00 - 8.00  U/L | 0.13 ± 0.35  (0.00 - 1.00) | 0.13 ± 0.35  (0.00 - 1.00) | 0.38 ± 0.74  (0.00 - 2.00) | 0.00 ± 0.00  (0.00 - 0.00) |  |
| Total Bilirubin | 0.00 - 0.20  mg/dL | 0.00 ± 0.00  (0.00 - 0.00) | 0.01 ± 0.04  (0.00 - 0.10) | 0.00 ± 0.00  (0.00 - 0.00) | 0.01 ± 0.04  (0.00 - 0.10) |  |
| Direct Bilirubin | 0.00 - 0.10  mg/dL | 0.00 ± 0.00  (0.00 - 0.00) | 0.00 ± 0.00  (0.00 - 0.00) | 0.00 ± 0.00  (0.00 - 0.00) | 0.00 ± 0.00  (0.00 - 0.00) |  |
| Indirect Bilirubin | 0.00 - 0.10  mg/dL | 0.00 ± 0.00  (0.00 - 0.00) | 0.01 ± 0.04  (0.00 - 0.10) | 0.00 ± 0.00  (0.00 - 0.00) | 0.01 ± 0.04  (0.00 - 0.10) |  |
| Amylase | 322.00 - 1310.00  U/L | 623.25 ± 98.12  (531.00 - 811.00) | 580.00 ± 79.06  (513.00 - 710.00) | 592.88 ± 64.55  (505.00 - 695.00) | 565.71 ± 102.25  (455.00 - 772.00) |  |
| Lipase | 15.00 - 228.00  U/L | 57.50 ± 25.86  (21.00 - 90.00) | 54.50 ± 22.98  (24.00 - 90.00) | 57.63 ± 27.72  (21.00 - 95.00) | 56.86 ± 26.79  (23.00 - 96.00) |  |
| Cholesterol | 136.00 - 392.00  mg/dL | 210.00 ± 23.63  (176.00 - 239.00) | 214.50 ± 26.35  (175.00 - 242.00) | 214.13 ± 30.98  (172.00 - 253.00) | 206.14 ± 32.13  (159.00 - 240.00) |  |
| Creatine Kinase | 64.00 - 314.00  U/L | 131.38 ± 53.71  (94.00 - 251.00) | 125.13 ± 24.39  (97.00 - 159.00) | 110.63 ± 13.65  (82.00 - 130.00) | 116.71 ± 15.57  (86.00 - 133.00) |  |
| ^1^Values are means ± standard deviation of the mean. Values in parentheses represent the range across all dogs (i.e., the minimum to the maximum value). | | | | | | |

| Supplemental Table 5. Chemistry profile on day 7 after oral administration of CBD isolate, CBDA isolate, CBDA FS and CBD/CBDA FS^1^ | | | | | |  |
| --- | --- | --- | --- | --- | --- | --- |
| Treatment |  | CBD isolate | CBDA isolate | CBDA FS | CBD/CBDA FS | |
| Items | Reference range | Day 7 | Day 7 | Day 7 | Day 7 | |
| Sodium | 143.00 - 150.00  mEq/L | 146.88 ± 1.46  (145.00 - 149.00) | 146.38 ± 1.60  (144.00 - 149.00) | 146.88 ± 0.99  (146.00 - 149.00) | 146.50 ± 1.07  (145.00 - 148.00) | |
| Potassium | 4.10 - 5.40  mEq/L | 4.83 ± 0.31  (4.30 - 5.20) | 4.94 ± 0.23  (4.60 - 5.40) | 4.89 ± 0.29  (4.70 - 5.50) | 4.95 ± 0.27  (4.60 - 5.40) | |
| Chloride | 106.00 - 114.00  mEq/L | 112.13 ± 3.04  (107.00 - 116.00) | 111.63 ± 1.51  (110.00 - 114.00) | 112.50 ± 1.51  (110.00 - 114.00) | 112.00 ± 1.07  (111.00 - 114.00) | |
| Bicarbonate | 14.00 - 24.00  mEq/L | 19.88 ± 1.73  (17 - 22) | 20.38 ± 1.51  (19.00 - 23.00) | 19.88 ± 1.46  (18.00 - 22.00) | 19.38 ± 1.69  (16.00.00 - 21.00) | |
| Urea Nitrogen | 9.00 - 26.00  mg/dL | 27.25 ± 5.12  (19.00 - 36.00) | 28.38 ± 2.20  (24.00 - 31.00) | 28.13 ± 3.27  (24.00 - 34.00) | 28.63 ± 1.51  (26.00 - 30.00) | |
| Creatinine | 0.60 - 1.40  mg/dL | 0.86 ± 0.13  (0.70 - 1.00) | 0.88 ± 0.07  (0.80 - 1.00) | 0.88 ± 0.07  (0.80 - 1.00) | 0.90 ± 0.05  (0.80 - 1.00) | |
| Calcium | 9.40 - 11.10  mg/dL | 10.51 ± 0.22  (10.20 - 10.80) | 10.54 ± 0.27  (10.20 - 11.00) | 10.56 ± 0.27  (10.20 - 11.1) | 0.90 ± 0.05  (0.80 - 1.00) | |
| Phosphate | 2.70 - 5.40  mg/dL | 5.44 ± 0.30  (5.10 - 5.80) | 5.28 ± 0.44  (4.60 - 6.00) | 5.49 ± 0.50  (4.60 - 6.20) | 5.34 ± 0.42  (4.70 - 5.90) | |
| Magnesium | 1.50 - 2.10  mEq/L | 1.63 ± 0.14  (1.40 - 1.90) | 1.59 ± 0.12  (1.40 - 1.80) | 1.63 ± 0.14  (1.40 - 1.80) | 1.63 ± 0.10  (1.50 - 1.80) | |
| Total Protein | 5.50 - 7.20  g/dL | 5.65 ± 0.23  (5.30 - 6.10) | 5.58 ± 0.09  (5.50 - 5.70) | 5.60 ± 0.30  (5.20 - 6.00) | 5.58 ± 0.22  (5.30 - 6.00) | |
| Albumin | 3.20 - 4.10  g/dL | 3.64 ± 0.21  (3.30 - 3.90) | 3.64 ± 0.12  (3.50 - 3.90) | 3.68 ± 0.09  (3.50 - 3.80) | 3.69 ± 0.12  (3.50 - 3.90) | |
| Globulin | 1.90 - 3.70  g/dL | 2.01 ± 0.22  (1.70 - 2.20) | 1.94 ± 0.18  (1.60 - 2.20) | 1.93 ± 0.27  (1.50 - 2.30) | 1.89 ± 0.21  (1.60 - 2.20) | |
| Glucose | 68.00 - 104.00  mg/dL | 83.75 ± 7.72  (78.00 - 101.00) | 85.75 ± 10.81  (66.00 - 96.00) | 86.00 ± 10.54  (76.00 - 109.00) | 90.00 ± 12.48  (67.00 - 104.00) | |
| ALT | 17.00 - 95.00  U/L | 26.00 ± 3.66  (23.00 - 34.00) | 26.88 ± 6.40  (19.00 - 41.00) | 27.13 ± 7.51  (20.00 - 43.00) | 28.13 ± 8.41  (21.00 - 47.00) | |
| AST | 18.00 - 56.00  U/L | 28.63 ± 3.74  (23.00 - 34.00) | 28.38 ± 4.50  (22.00 - 38.00) | 27.38 ± 5.50  (22.00 - 37.00) | 27.63 ± 4.47  (23.00 - 37.00) | |
| Alkaline Phosphatase | 7.00 - 115.00  U/L | 61.00 ± 37.95  (25.00 - 137.00) | 68.88 ± 65.18  (27.00 - 223.00) | 45.88 ± 14.80  (23.00 - 70.00) | 51.00 ± 19.73  (26.00 - 92.00) | |
| GGT | 0.00 - 8.00  U/L | 0.38 ± 0.74  (0.00 - 2.00) | 0.00 ± 0.00  (0.00 - 0.00) | 0.13 ± 0.35  (0.00 - 1.00) | 0.25 ± 0.71  (0.00 - 2.00) | |
| Total Bilirubin | 0.00 - 0.20  mg/dL | 0.01 ± 0.04  (0.00 - 0.1) | 0.01 ± 0.04  (0.00 - 0.10) | 0.00 ± 0.00  (0.00 - 0.00) | 0.00 ± 0.00  (0.00 - 0.00) | |
| Direct Bilirubin | 0.00 - 0.10  mg/dL | 0.00 ± 0.00  (0.00 - 0.00) | 0.00 ± 0.00  (0.00 - 0.00) | 0.00 ± 0.00  (0.00 - 0.00) | 0.00 ± 0.00  (0.00 - 0.00) | |
| Indirect Bilirubin | 0.00 - 0.10  mg/dL | 0.01 ± 0.04  (0.00 - 0.10) | 0.01 ± 0.04  (0.00 - 0.10) | 0.00 ± 0.00  (0.00 - 0.00) | 0.00 ± 0.00  (0.00 - 0.00) | |
| Amylase | 322.00 - 1310.00  U/L | 608.00 ± 80.28  (493.00 - 741.00) | 557.25 ± 65.52  (499.00 - 686.00) | 589.63 ± 58.41  (521.00 - 680.00) | 585.75 ± 68.02  (489.00 - 685.00) | |
| Lipase | 15.00 - 228.00  U/L | 56.00 ± 26.55  (20.00 - 88.00) | 55.25 ± 22.58  (25.00 - 81.00) | 59.13 ± 27.81  (22.00 - 90.00) | 55.75 ± 23.52  (23.00 - 87.00) | |
| Cholesterol | 136.00 - 392.00  mg/dL | 228.25 ± 25.24  (197.00 - 266.00) | 229.25 ± 26.06  (197.00 - 272.00) | 235.25 ± 26.04  (197.00 - 272.00) | 230.88 ± 22.99  (193.00 - 264.00) | |
| Creatine Kinase | 64.00 - 314.00  U/L | 128.88 ± 20.86  (107.00 - 166.00) | 159.63 ± 83.41  (83.00 - 338.00) | 125.88 ± 22.00  (107.00 - 162.00) | 114.50 ± 8.91  (97.00 - 125.00) | |
| ^1^Values are means ± standard deviation of the mean. Values in parentheses represent the range across all dogs (i.e., the minimum to the maximum value). | | | | | |  |

Supplemental Table 6. Heart rate in dogs 1- and 4- hour post administration with CBD isolate, CBDA isolate, CBDA full spectrum and CBD/CBDA full spectrum^1^.

|  | CBD isolate | | | | | | | | CBDA isolate | | |
| --- | --- | --- | --- | --- | --- | --- | --- | --- | --- | --- | --- |
| Dog name | baseline heart rate | 1 hour post dosing heart rate | 4-hour post dosing heart rate | | baseline heart rate | | | 1 hour post dosing heart rate | | 4-hour post dosing heart rate | |
| Roy | 92 | 100.00 ± 5.66 | 99.00 ± 4.24 | | 96 | | | 98.00 ± 8.49 | | 98.00 ± 0.00 | |
| Dougal | 104 | 112.00 ± 5.66 | 100.00 ± 0.00 | | 104 | | | 103.00 ± 4.24 | | 104.00 ± 2.83 | |
| Murtagh | 92 | 109.00 ± 1.41 | 106.00 ± 8.49 | | 108 | | | 109.00 ± 4.24 | | 108.00 ± 2.83 | |
| Jamie | 96 | 109.00 ± 4.24 | 110.00 ± 0.00 | | 106 | | | 107.00 ± 4.24 | | 108.00 ± 5.66 | |
| Bex | 100 | 100.00 ± 5.66 | 101.00 ± 1.41 | | 114 | | | 104.00 ± 8.49 | | 103.00 ± 7.07 | |
| Keeley | 96 | 103.00 ± 7.07 | 105.00 ± 7.07 | | 106 | | | 101.00 ± 7.07 | | 106.00 ± 8.49 | |
| Marion | 102 | 106.00 ± 2.83 | 104.00 ± 5.66 | | 80 | | | 96.00 ± 2.83 | | 94.00 ± 2.83 | |
| Mae | 96 | 104.00 ± 0.00 | 104.00 ± 5.66 | | 72 | | | 93.00 ± 9.90 | | 97.00 ± 1.41 | |
|  |  | | | | | | | |  | | |
|  | CBDA full spectrum | | | | | | | | CBD/CBDA full spectrum | | |
| Dog name | baseline heart rate | 1 hour post dosing  heart rate | | 4-hour post dosing heart rate | | baseline heart rate | 1 hour post dosing heart rate | | | | 4-hour post dosing heart rate |
| Roy | 96 | 98.00 ± 8.49 | | 98.00 ± 5.66 | | 108 | 105.00 ± 12.73 | | | | 103.00 ± 7.07 |
| Dougal | 104 | 104.00 ± 5.66 | | 104.00 ± 2.83 | | 100 | 105.00 ± 1.41 | | | | 113.00 ± 1.41 |
| Murtagh | 102 | 99.00 ± 1.41 | | 100.00 ± 0.00 | | 96 | 102.00 ± 0.00 | | | | 101.00 ± 4.24 |
| Jamie | 98 | 102.00 ± 11.31 | | 105.00 ± 1.41 | | 76 | 103.00 ± 4.24 | | | | 99.00 ± 7.07 |
| Bex | 98 | 105.00 ± 12.73 | | 109.00 ± 7.07 | | 108 | 106.00 ± 2.83 | | | | 102.00 ± 0.00 |
| Keeley | 108 | 113.00 ± 12.73 | | 109.00 ± 1.41 | | 110 | 104.00 ± 2.83 | | | | 104.00 ± 5.66 |
| Marion | 110 | 110.00 ± 0.00 | | 105.00 ± 1.41 | | 112 | 105.00 ± 7.07 | | | | 107.00 ± 7.07 |
| Mae | 98 | 104.00 ± 5.66 | | 100.00 ± 0.00 | | 92 | 103.00 ± 1.41 | | | | 102.00 ± 2.83 |

^1^Values are means ± standard deviation.
